# Public perspectives on COVID-19 public health and social measures in Japan and the United Kingdom: a qualitative study

**DOI:** 10.1186/s12889-024-18866-3

**Published:** 2024-05-23

**Authors:** Saki Kawamitsu, Tin Zar Win, Su Myat Han, Tomoka Nakamura, Melissa Jogie, Chris Smith

**Affiliations:** 1 School of Tropical Medicine and Global Health, Nagasaki University, Nagasaki, Japan; 2 Department of Infectious Disease Epidemiology, Faculty of Epidemiology and Population Health, London School of Hygiene and Tropical Medicine, London, UK; 3 University of Roehampton, London, UK; 4 Department of Clinical Research, London School of Hygiene and Tropical Medicine, London, UK; 5 Present Address: School of Health Sciences, Faculty of Medicine and Graduate School of Health Sciences, University of the Ryukyus, Okinawa, Japan; 6 Present Address: National Centre for Infectious Disease, Novena, Singapore

**Keywords:** COVID-19, SARS-CoV-2, Public health and social measures, Japan, United Kingdom, England, Pandemic preparedness, Focus groups

## Abstract

**Background:**

The COVID-19 pandemic, caused by SARS-CoV-2, was one of the greatest modern public health crises that the world has faced. Countries undertook sweeping public health and social measures (PHSM); including environmental actions such as disinfection and ventilation; surveillance and response, such as contact tracing and quarantine; physical, such as crowd control; and restrictions on travel. This study focuses on the public perceptions of PHSM in two countries, Japan and the United Kingdom (UK) as examples of high-income countries that adopted different measures over the course of the pandemic.

**Methods:**

This study was conducted between November 2021 and February 2022, a period in which the Omicron variant of SARS-CoV-2 was predominant. Fourteen online focus group discussions were conducted in each country. Overall, 106 total participants (50 from the UK and 56 from Japan) participated in 23 focus groups (11 in the UK and 12 in Japan) with an average of three to six participants per group. Both countries were compared using a thematic analysis method.

**Results:**

Both countries’ participants agreed that vaccination was an effective measure. However, they did not favor mandatory vaccination policies. Working from home was well accepted by both sides, but they reported that schools should have continued to be opened as before COVID-19. Both sides of participants expressed that temperature testing alone in indoor facilities was ineffective as a COVID-19 control measure. There were contrasting views on face covering rules in public spaces, international and domestic movement restrictions. High acceptance of mask-wearing was reflective of Japanese customs, while it was accepted as a strong recommendation for participants in the UK. Japanese participants favored quarantine for international travel, while the UK participants supported banning non-essential travel.

**Conclusion:**

Similar and contrasting views on PHSM against COVID-19 between Japan and the UK demonstrated how policies in controlling an epidemic should be tailored by country with respect to its norms, cultures, economic and disease burden. Our findings may guide how policy makers can engage with the public through effective health communication and consider regulations that are aligned with the public’s views and capacities in changing their behavior for future pandemic preparedness.

## Introduction

The COVID-19 pandemic caused by SARS-CoV-2 was one of the greatest public health crises the world has faced, and in response countries undertook considerable public health and social measures (PHSM). PHSM is defined by the World Health Organization (WHO) as measures or actions by individuals, institutions, communities, local and national governments, and international bodies to slow or stop the spread of infectious disease, such as COVID-19 [1]. PHSMs include but are not limited to vaccination policies, face covering rules, working and teaching hours for businesses and schools, testing requirements to access indoor events, and international and domestic travel restrictions. The adoption of PHSMs was the subject of much debate during the COVID-19 pandemic [2]. Although all countries aimed to achieve the same outcome – to stop or dampen the spread of the diseases and death without burdening their health resources and economic vigor – there was a recognition that there exists no one-size fits all policy.

In 2021 the British Academy funded a small portfolio of projects focusing on vaccine engagement across the G7 countries [3]. The funded project; Adapting to the ‘New Normal’: Implications for post-COVID-19 Health Communication and Education [4], specifically focused on Japan and the United Kingdom (UK) as the only G7 island nations. Despite with similar constitutional governments, these nations were influenced by distinct socio-cultural and economic factors. This allowed for a comparative analysis of population responses to public health measures across diverse social-cultural settings but under similar government policy frameworks.

In align with this, the current study focused on Japan and the United Kingdom (UK), countries that adopted different PHSM over the course of the pandemic; albeit with slight variations within different prefectures of Japan and regions of the UK. Both Japan and the UK are islands, high-income countries, with similar demographic profiles including large proportions of elderly in their populations. The study aimed to identify why public voices should be considered when designing long-term plans for PHSMs to help prepare for future pandemics, and to identify culturally specific traits of populations as displaying homogenous behaviors. This could help with the curation of messages in terms of knowing when and how to approach the public about policy changes.

## Methods

### Study setting and study participants

The funded project; Adapting to the ‘New Normal’: Implications for post-COVID-19 Health Communication and Education used a mixed methods design; (i) survey with experimental design and (ii) focus groups. The discrete choice experiment aimed to assess the cost-benefit preferences the public would make while the focus groups provided a sample of qualitative insights creating a rationale for making these choices. While the large-scale survey provided patterns of choices, the focus groups were important to complement the survey as it helped us understand the rationale behind these choices.

In this study, we utilized the mixed-gender focus group discussions (FGDs) part only with the aim of providing insights into the public assessment and understanding of the advantages and disadvantages that individuals may envision when considering COVID-19 public health and social measures (PHSMs). Six PHSM categories were chosen (Table 1) for the FGDs. The discussions were held online, because the study was conducted when the Omicron variant of SARS-CoV-2 was predominant, and the prevailing COVID-19 preventive measures prohibited group gatherings.

The participants were recruited through snowball sampling and online platforms (Facebook, Twitter, and website introducing the project) by purposive convenience sampling between November 2021 and February 2022. The study obtained information on age, gender, ethnicity, residence, occupation, the number of COVID-19 vaccinations that they had received and specific dates and times if and when they could participate for focus group discussions. Individuals aged more than 18 years old were eligible for FGDs, if they lived in the Kansai region (Japan) or Greater London (the UK). The Kansai region is located on the west side of Japan and consists of six prefectures: Osaka, Hyogo, Kyoto, Nara, Wakayama, and Shiga. Approximately 20.4 million people lived in the region, which comprised 16.7% of the total population of Japan in 2021 [5]. Osaka is the second largest city after the capital of Tokyo. The population and businesses are mainly dispersed among the three major cities of Osaka (Osaka prefecture), Kobe (Hyogo prefecture), and Kyoto (Kyoto prefecture). Greater London is the administrative area of London, the capital of the United Kingdom and England. It is organized into 33 local administrative divisions, consisting of 32 London boroughs and the City of London. The population of Greater London was approximately 9 million in 2021 [6]. The researchers then grouped the participants according to the available dates and times so that they were as mixed in age and gender as possible, in anticipation of the group dynamics that would emerge from interactions among participants with diverse backgrounds.

### Data collection

Online focus groups were conducted separately in Japan and the UK. The focus group topic guides included questions on the six main categories of PHSMs: (1) vaccination; (2) face covering rules; (3) working and teaching hours for businesses, schools, and universities; (4) testing required to access indoor events; (5) domestic movement restriction; and (6) border closure and international travel restrictions. The participants were asked to select the level of control measures for each category according to the type of COVID-19 scenarios that differed in epidemiological profiles with varying cases, death rates, and hospitalization trends (Table 1). As these scenarios changed, the participants were asked anew whether they would alter their preference level of measures to adapt to these changes. This process provided participant views of PHSMs responding to ‘shocks’, or sharp changes in the headline levels of infections, hospitalizations, and deaths. The epidemiological profile of COVID-19 scenarios was based on (i) the number of new cases per million people per week (ranging from 200 to 4000), (ii) the percentage of excess deaths per month (ranging from − 10 to 25%), and (iii) the overall trend in the number of hospitalizations over the previous 2 weeks (either ‘rising’ or ‘falling’). The participants were asked to explore the reasons for the selections in each phase. Based on the principle of focus group design, the PI was able to customize a design for the focus group integrated within the larger mixed-method design of the study [7]. The focus groups used similar prompts and references of the scenarios in the survey to elicit participants’ detailed thought processes and choices, enhancing the validity of the overall mixed-methods design. Each group discussion lasted between 100 and 120 min.

The discussion guide was initially created in English, and then it was translated into Japanese to fit the Japanese context. A pilot test was conducted to scrutinize the content of the discussion guide. Before starting the data collection, the principal investigator (PI) conducted training sessions for the research assistants (RAs) on how to conduct the focus group discussions (FDGs). Specifically, the moderators were trained to ask participants individually and by name for their thoughts (promoting inclusivity), and to defuse any political contexts. Participants were given the option to turn the camera on or off, but were encouraged to keep them on as much as possible to allow for the observation of facial expressions. The study addressed the possibility of bias in data collection by training moderators to ensure that participants responded using their own words and phrases, and elaborated on their thoughts independently before being prompted by researchers.

In each country, the PI and/or the RAs moderated the group discussions. Each focus group had two researchers; specifically, one was mainly a moderator who ensured the smooth progression of the sessions and note taking, while the other was mainly an observer who made sure that all of the topics and questions were covered. The observer was responsible for recording the time, providing technical support, observing remarks and facial expressions, note taking, and had decision-making authority in the event of a tie in the number of votes. Each focus group conducted was moderated by native Japanese speaker in Japan and by native English speaker in the UK .

Overall, 106 participants were recruited and participated in the FDGs. In Japan, 56 participants participated in 12 FGDs, with an average of three to six participants per group. They were conducted between 8th January 2022 and 12th February 2022.

In the UK, 50 participants participated in 11 focus groups, with an average of three to six participants per group. FGDs in the UK were conducted between 28th December 2021 and 21st January 2022.

**Table 1:** Public health and social measures (PHSM) categories and level options

PHSM Category	Level	Description
Vaccination policy (National)	**1**	General information campaign, no penalties if unvaccinated.
	**2**	Vaccine strongly advised and limited service if unvaccinated.
	**3**	Vaccines compulsory for everyone.
Face covering rules in public spaces	**1**	Face covering rules in public spaces, and recommended only, not forced.
	**2**	Mandatory fines for non-compliance.
Working and teaching hours for businesses and schools	**1**	Regular (maintains economy).
	**2**	Minimal (relieves health services).
Testing required to access indoor events	**1**	Temperature checks (easy but unreliable).
	**2**	Lateral flow/antigen (uncomfortable but more reliable).
International travel restriction	**1**	Fewer/limited flights (but no quarantine).
	**2**	Frequent/regular flights (but long quarantine).
	**3**	Bans on all non-essential entry and exit.
Domestic movement restrictions	**1**	Overnight curfew (stay indoors between 9 pm and 6 am).
	**2**	Commuting limited to local town, city or prefecture.

### Data analysis

All recorded video and audio were transcribed by NVivo transcription software and checked by RAs. Japanese FGD transcripts were translated into English by the RAs. To ensure data consistency, this study had two researchers in each session to compare each note and recorded data for the accuracy and consistency in the data collected. Additionally, to ensure data reliability, this study introduced variations in the scenarios to assess the participants’ consistency in their choices. The transcripts were read multiple times to develop a deeper understanding of the data. Then, thematic analysis was used to analyze the data and present the results according to the main themes that emerged together with illustrative quotes.During the analysis phase, discussions were held between native speakers of Japanese and English to capture the nuances of the speakers and the cultural background necessary to interpret and discuss the results of all focus groups conducted in Japan and the UK.

## Results

Characteristics of the participants in the focus group discussions are shown in Table 2. Findings from the FGDs in Japan are reported first, followed by the UK.

**Table 2:** Characteristics of participants

Characteristics	United Kingdom (*n* = 50)	Japan (*n* = 56)
**Age**		
18–39	36	42
40–59	13	11
60 and above	1	3
**Sex**		
Female	26	27
Male	24	29
**Occupation**		
Financial	1	0
Health service	5	3
Consultant	0	0
Services	3	2
Others	41	51
**Vaccination**		
None	5	5
1 dose	6	0
2 doses	7	51
3 doses 4 doses	19 13	- -

### Response to COVID-19 preventive measures in Japan

In general, Japanese participants mainly emphasized the number of cases and hospitalizations rather than the number of deaths. There was a preference to maintain restrictions regardless of the number of cases, because they expected that the numbers would increase again. Many participants recognized the economic damage and agreed that economic activities should be prioritized when the number of cases decreased.

#### Vaccination is an effective measure but should not be mandatory

The majority of Japanese participants believed in the effectiveness of the vaccine; but even under high infectious scenarios, they opposed making vaccination mandatory, in consideration of respect for human rights and the differing situations of individuals. Although 60% of participants chose the option of strongly recommending against limiting services to the unvaccinated, they preferred to make advantages for those who were vaccinated instead of imposing penalties or restrictions on those who were unvaccinated.

*“We need to guarantee individual freedom, so I chose Level 2 (Vaccine strongly advised and limited service if unvaccinated). Rather than restricting services to the unvaccinated, I thought that vaccination would go more smoothly if there were benefits to those who had been vaccinated.” (Female, 18–39 s, FG2)*.

However, in the scenario that cases decreased, some participants who had experienced adverse reactions to vaccination reported preferring a general information campaign versus mandatory vaccination.

*“I choose level 1(General information campaign, No penalties if unvaccinated). I had a very strong side effect from the vaccine, and my fever was not so bad, about 38 degrees Celsius, but I felt muscle pain so much that I was bedridden for about three days. Since I know the situation, I think that if more and more people get vaccinated twice, they will probably ask for a third and fourth vaccination. If it became a requirement and I was restricted from doing many things, I would not be happy.” (Female, 18–39 s, FG5)*.

#### Quarantine as an effective measure to control imported cases

Around 60% of participants in Japan believed that COVID-19 was repeatedly brought in from outside the country; and because of the effectiveness of quarantine, more emphasis should be placed on the quarantine period for international travel under high infectious scenarios.

*“People are coming from overseas anyway. Even if there are restrictions on non-essential overseas travel, people will enter the country even if they don’t need to, so it is better to have a quarantine period.” (Male, 40–59 s, FG11)*.

In the scenario that COVID-19 became stable, such as under low infectious scenarios, many participants reported preferring to have frequent or regular flights which provided for a quarantine period, because a total ban on unnecessary international travels was impossible given the economic damage.

*“I have a very similar opinion to the person who just said, and that is level 2 (Frequent/regular flights (but long quarantine)). I think it is a compromise between the two. I think that setting a quarantine period will lead to a decrease in unnecessary travel, such as travel for entertainment and sightseeing. We can’t eliminate such things. If we focus on the effect of drastically restricting such activities, I think this is better.” (Male, 18–39 s, FG5)*.

#### Domestic travel restrictions play a role in reducing the spread of infection

Many people in Japan thought that under high infectious scenarios, commuting limited to local towns, cities, or prefectures would be appropriate because the number of cases was large; and activities should be restricted during the daytime, when there was a lot of human activity. However, some thought that due to the economic impact, and based on their experience, restricting activities during the daytime would be too severe.

*“…domestic travel should be restricted during the day. The number of infected people is high, exceeding 10,000. It depends on the virulence of the virus, but it is important not to spread the infection. The infection has spread without restrictions during the day.” (Male, 18–39 s, FG10)*.

The participants reported that they might change their preference if the severity of the COVID-19 pandemic were to reduce. Around 50% of participants reported that they may choose an overnight curfew, while the remaining chose commuting limited to local town, city, or prefecture. It was mentioned that it would be difficult to limit movement of people in the Kansai area, where people frequently come and go from neighboring prefectures for commuting to work and school.

*“Within Kansai area is close to neighboring prefectures, and many people commute to school and work across the region, so level 2 (Commuting limited to local town, city or prefecture) is difficult.” (Female, 18–39 s, FG6)*.

#### Work from home is an appropriate measure but may not be good for schools

Most participants in Japan agreed that under high infectious scenarios businesspeople and companies should use telework to minimize direct human contact. However, they believed that schooling should be continued, especially for elementary and junior high schools, as virtual learning could impact student social skills, education, and physical activity.

*“Even if students take online classes, it’s not good for their health if they stay at home all the time and don’t do any physical activity. School is also important for social skills, so I would like to make it level 1(Regular (maintains economy)) to respect the right of children to learn.” (Female, 18–39 s, FG2)*.

Participants reported that if the number of cases decreased, regulations should be loosened by accepting the presence of COVID-19 as the new normal, and schools should be reopened, considering the importance of student education.

*“I think it would be good to weaken the restrictions on working/schooling. As everyone mentioned earlier, if commuting is restricted, I think the situation will change to the new normal where people will be able to live with this. If the COVID-19 situation is reduced to this level, I think that schools should return to normal, and everyone should be able to study to some extent.” (Female, 18–39 s, FG4)*.

#### Temperature checks alone are not a sufficient measure for indoor events

Almost all participants had the opinion that in their experience a temperature check alone was not effective. Most participants supported lateral flow or antigen testing due to its reliability.

*“I also don’t trust the temperature check alone, so I think the antigen test is more reliable. I think the more checks you do, the more likely you can find people who are positive.” (Female, 60s, FG12)*.

In the scenario that cases decreased, most participants reported that they would accept the use of temperature screening, in consideration of the financial costs, human resources, and time taken to implement antigen testing.

*“The number of cases has decreased significantly, and the number of hospitalizations has gone down from the previous increase, so I am imagining the last part of a wave that came once. As for the number of deaths, I don’t feel that there is a significant difference between 9% fewer and 3% more deaths, so I’m thinking that we should loosen up the measures. I think vaccination is fine, but I don’t think we need to spend so much money and time on antigen testing for events. I think it’s also the right time to ensure people’s freedom of movement without setting quarantine periods for restrictions on overseas travel.” (Male, 40–59 s, FG11)*.

#### Mask wearing is a custom in Japan and an effective measure

All participants thought that wearing masks in public spaces should be recommended rather than forced, because almost all people in Japan wear masks, and new measures would not likely increase the rate of the mask use. They believed that wearing masks was important and effective in preventing infection.

*“Considering the cost of establishing such laws and regulations, I thought it would be fine to leave it as it is, because all Japanese people are currently wearing masks.” (Male, 18–39 s, FG3,)*

*“I think people will wear masks just because it is cultural. If you look at the U.S., Europe, and other countries, you will find that there are many people who do not wear masks. In Japan, it is not compulsory to wear masks, and even if there is no fine, people would probably wear masks in public places, and I think people can cooperate in wearing masks even if there are no strict rules.” (Female, 18–39 s, FG9)*.

### Response to COVID-19 preventive measures in the UK

Most participants made decisions based on the hospitalization and death rate rather than the number of cases.

#### Vaccination is an effective measure but should not be mandatory

As in Japan, participants in the UK did not recommend compulsory vaccination, out of consideration for human rights. But many participants were in favor of limiting services for the unvaccinated.

*“I would go with level 2 (Vaccine strongly advised and limited service if unvaccinated) as well. Also, for this reason I don’t think it should be compulsory to have vaccines, however if it is strongly advised and it’s your choice not to have it then the consequences of you not having it affect what you can do.” (Female, 40–59 s, FG2)*.

*“To keep such a good condition, I think a vaccine is necessary, but not mandatory because there must be someone who is concerned not to have the vaccine and they can still keep their freedom.” (Female, 40–59 s, FG3)*.

#### Limiting international travel to only essential trips may reduce the spread the viruses

Around 50% of participants reported preferring frequent or regular flights with long quarantine times; whilst the other half preferred a government ban on all non-essential international travel, with the expectation that the policy would impede the entry of new variants into the country.

*“I would also choose level 3 (bans on all non-essential entry and exit). Just like [name removed] had said, there are people carrying viruses from other countries. So, I think, in regard to the case study, I think if we ban all non-essential entries and exits, then hopefully that’ll crack down on any additional new variants.” Female, 18–39 s FG6)*

#### Domestic travel restrictions do not have any impact on control measures

Most of the participants did not see any difference between an overnight curfew and limited commuting. They thought that both options still allowed people to contact each other.

*“I think what we’d want to do is reduce contact as much as possible, so with the third kind of question with commuting limited to local towns versus not seeing each other, the curfew after hours, those two again I don’t feel strongly about because they don’t really make a difference. You’re still seeing people either way. I guess still staying with the commuting within a limited local town reduces it from spreading to another geographical area.” (Female, 18–39 s, FG9)*.

#### Limited working hours is effective, but it may not be a good choice for the long term

Roughly 60% of the participants believed that activities such as schools, universities, and business should be reduced; as they could increase the number of cases and hospitalizations due to close contact. However, some participants were concerned about mental health issues and domestic violence resulting from isolation at home.

*“You understand, when you reduce the number of times in school and businesses, people will not be in contact, hence the reduction in the number of people who would be going to hospital.” (Male, 18–39 s, FG11)*.

*“At the same time, I’ve also noticed within my profession that domestic abuse has risen and mental health has risen, and people have taken their lives and people have been very hurt in domestic abuse situations. So, that’s the only reason I would go with regular.” (Female, 18–39 s, FG1)*.

#### Temperature checks alone are not a sufficient measure for indoor events

Around 70% of participants preferred lateral flow or antigen testing, as they believed that a temperature check alone was not reliable. However, it was reported that lateral flow or antigen tests may be uncomfortable for some people.

*“For me, I’d say level 2 (Lateral flow/antigen (uncomfortable but more reliable)) because it is a little uncomfortable but to get reliable data is quite important.” (Male, 18–39 s, FG4)*.

*“I would definitely choose level 1 (Temperature checks (easy but unreliable)). Because it’s easy and I don’t see if lateral flow or antigen tests can be comfortable for everyone.” (Male, 18–39 s, FG4)*.

#### Mask wearing prevents transmission but recommendation alone is not effective to public behavior changes

The majority of participants (88%) supported mandatory fines for non-compliance, since they were concerned that recommendations alone may be insufficient to change behaviors, and trusted that masks could reduce transmission due to a respiratory tract infection. However, there were disagreements about human rights if the government made mask-wearing a mandatory measure.

*“I have somebody that I know that is from China background and they said that even before COVID, they’ve always had to wear masks in public transport, and they don’t really get much colds and flu’s anyway. Yes, I’ll go with level 2 (Mandatory fines for non-compliance).” (Female,18–39 s, FG1)*.

*“I think based on the data above that there’s a lot of cases and hospital admissions are rising, I think I would opt for level 2 (Mandatory fines for non-compliance) because experience told me when it’s recommended then most people don’t follow. But if you have to then you may get slightly more people follow the rules.” (Female, 40–59 s, FG2)*.

Table 3 describes the overall similarities and differences in the responses of participants to the selected PHSM of COVID-19.

**Table 3:** Summary of participants responses to PHSM categories and level options

PHSMs	Similarities between the UK and Japan	Differences	
		United Kingdom	Japan
**Vaccines**	All participants did not agree to enforce the level 3 option: “Vaccines compulsory for everyone” (all case scenarios). Participants preferred either level 1: “General information campaign, No penalties if unvaccinated”; or level 2: “Vaccine strongly advised and limited service if unvaccinated” (all case scenarios).		
**International travel**		Participants chose level 3: “Bans on all non-essential entry and exit” on all case scenarios	Participants preferred the level 2 option: “Frequent/regular flights (but long quarantine)
**Domestic travel restriction**		Participants do not see domestic restrictions or curfews as effective. They believe people will have contact in some ways even with the restrictions.	Participants chose the level 1 option during the low number of cases of COVID-19. Participants chose the level 2 option when the case load is high.
**Working/teaching hours for business/ schools**	Both countries had in common the encouragement of working from home or teleworking, depending on the type of work. Participants accepted level 2: “minimal hours” (or work from home or telework). (all scenarios)	The perspectives mainly focus on minimizing contact, encouraging people to reduce interaction in business settings.	The perspectives mainly concern the negative impact of school closures on children. Participants reported that schools should be reopened to ensure the continued education of students.
**COVID-19 testing in indoor events**	Participants reported that level 1 “temperature check alone” was not effective in high case scenarios	Participants chose the level 2 option “lateral flow/antigen testing”, as they believe that testing temperature alone was not effective or reliable.	Participants chose level 1 (temperature check alone) during the low cases of COVID-19. But they chose level 2 “lateral flow/antigen testing” during the high case scenario.
**Masks**		The participants chose level 2: “Mandatory fines for non-compliance”.	The participants reported that mask wearing is well accustomed in Japan and thus level 1 “recommendation for mask-wearing” is enough. They believed forced policy of mask wearing will not increase the already high rate of mask wearing.

## Discussion

In this study we conducted focus group discussions with 106 people in Japan and the United Kingdom (UK), to investigate public perceptions of levels of COVID-19 prevention measures under different hypothetical degree scenarios of the pandemic. The study spanned from late 2021 to early 2022, a time at which the Omicron variant of SARS-CoV-2 was predominant. To the best of our knowledge, this is the first comparative study of its kind.

In the FGDs, participants in the UK judged the level of countermeasures based on the number of deaths and hospitalizations, while those in Japan focused on the number of cases and hospitalizations. There were similarities and differences between Japanese and the UK perspectives on different PHSMs.

### Vaccines

Most participants in both countries accepted the strong recommendation for vaccination, and limiting services to the unvaccinated. However, a collective resistance to mandatory vaccination persisted across all conceivable COVID-19 scenarios. These findings were consistent with a discrete choice survey conducted in the USA, which explored preferences for strategies related to COVID-19 vaccine distribution [8]. The most common reasons against mandatory vaccination were human rights and the right to freedom of choice, and also considering those who were physically unable to be vaccinated. The perceptions of participants from both countries on rewarding the vaccinated were in line with a study in the Netherlands in which respondents particularly disliked the policies penalizing those who abstain from vaccination, while favoring approaches that reward vaccine acceptance [9]. The opposition to mandatory vaccination may be in consideration of human rights and the preservation of individual freedom of choice; as well as in recognition of those who had legitimate medical reasons for being ineligible for vaccination. Within the UK, the issue of mandated COVID-19 vaccination was a divisive one, leading to a polarization of public sentiment [10]. It is crucial to recognize that mandates and restrictions carry profound ethical implications [11]; and possess the potential to elicit a strong and often negative public reaction [12, 13].

The high acceptance of COVID-19 vaccination observed in our study is likely attributed to the widespread recognition that vaccination is the foremost efficacious measure for curtailing the incidence of COVID-19 cases and mitigating hospitalizations. It was reported that 64–70% in the UK [14], and 56–62% of individuals in Japan [15], had COVID-19 vaccine confidence; and the acceptance is related to the high effectiveness of the vaccine during the time of the study [16, 17]. Positive and pervasive media attention regarding the effectiveness of the vaccine may have influenced opinions; particularly in Japan where the government or media is perhaps the main source of information [8]. However, the participants suggested that there is a room for improvement in the transparency and clarity of government health communications to the public.

### Travel restrictions

Both countries are geographically islands, and this might have influenced the shared concerns regarding the implementation of international and domestic travel restrictions. Participants in both countries recognized the importance of quarantine periods. Due to the economic impact of flight bans, most Japanese respondents focused on the quarantine system; while about half of respondents in the UK preferred to ban all non-essential travel, as they thought that every entry could bring the virus, or a new variant. It was estimated that in tourism revenue, Japan could lose 1.29 billion USD during the first quarter of 2020 [18]; and the UK could lose £7 billion during the Omicron pandemic [19]. Although both countries suffered from the economic impact, differences in participant responses could have been influenced by their governments’ response to the pandemic and the current COVID-19 situation in their countries. It is worth noting that in Japan, a national lockdown is not possible by law, and therefore the willingness of the public to adhere to suggestions was considered important for the flattening of the COVID-19 curve [12]. In contrast, in the UK, a national lockdown required residents to stay home unless there was an essential need to go out. Public business activities may have a large impact on the behaviors of individuals.

### Working hours

Individuals from both countries were adapting to new ways of teleworking under COVID-19 measures. They were in favor of continuing remote work situations beyond the conclusion of the pandemic. In the UK, telework was mainly discussed, with support for its introduction and minimizing the use of public transportation to reduce human contact as an essential infection control measure. On the other hand, in Japan, schooling was mainly discussed rather than telework. Participants expressed concerns about the negative impact of school closures on children’s development and stressed the importance of schooling, even if it slightly increases the risk of infection, assuming that other policies such as vaccines are in place. Thisdifference could potentially be attributed to the relatively youthful composition of the Japanese participants, coupled with the potential challenges in effectively instituting online learning at the time of data collection comparing to the UK situation.

### COVID-19 testing

Participants from both countries did not agree that temperature screening alone was an effective method for identifying suspected cases, especially when the COVID-19 cases were high, as was the case at the time of the study. Previous studies [20–22] supported this response, stating that temperature screening methods alone should not be the sole measure for case detection. A systematic review and meta-analysis found that 40–50% of confirmed COVID-19 cases were asymptomatic [23]; thus, perhaps undermining the reliability of temperature checks as a diagnostic tool. However, Japanese participants accepted the idea of taking temperature readings only, in the scenario with low number of COVID-19 cases; as they assumed that the lateral flow tests or polymerase chain reaction (PCR) tests required significant financial, human, and time resources.

### Mask wearing

Participants from both countries acknowledged the effectiveness of wearing masks in preventing infection. In Japan, the participants indicated that there was no need for such regulations, citing the longstanding Japanese custom of wearing masks [24]. In contrast, the participants from the UK advocated for stricter regulation on mask-wearing, as the population is not as accustomed to this practice as in Japan. In 2020, approximately 80% of people in Japan wore masks to prevent COVID-19 transmission [25]. The cultural emphasis on self-restraint to curb the spread of infection may have contributed to the high compliance rate for mask-wearing as a control measure against COVID-19. Given the widespread acceptance of mask-wearing, the general population in Japan and in other Asian countries may have been strongly motivated to adhere to policies and guidelines that encourage the wearing of face masks in public spaces to combat the COVID-19 pandemic [26].

### Strengths and limitations

This study captured the real-time opinions during the period of the Omicron pandemic, when infection levels and concerns about new variants were high. Although public opinions were dynamic, our findings retained significance as a historical record, and reflected individual viewpoints within the context of the COVID-19 pandemic. These insights could prove valuable to policymakers when contemplating hypothetical scenarios for future COVID-19 re-emergence or outbreaks of other pathogens.

Our study had some limitations. First, the study was conducted online, to avoid gathering people under COVID-19. This left us with online recruitment of participants, and convenience sampling restricted the group to only those with internet access and the capacity to engage in online interactions; and this was particularly noticeable among the younger participants in Japan. Second, the study was conducted during a specific period where the preferences of participants may have depended on their availabilities and the epidemiological situation when a new SARS-CoV-2 variant had just begun to circulate. Third, data collection occurred during a transitional phase in both countries and spanned a period of evolving infection scenarios and response strategies. Despite the guidance of the moderator to anchor responses to the hypothetical scenarios rather than current circumstances, the infection conditions at the time of the FGDs may still have influenced perceptions and replies. Fourth, inherent bias could arise from the likelihood of participation being skewed toward individuals interested in COVID-19 control measures; and leaving out the opinions of those entirely disinterested or those who may delineate from the views of the majority. Finally, there were slight variations to PHSMs within different prefectures of Japan and regions of the UK. Hence the public perspectives obtained from the Kansai and Greater London areas may not be representative those throughout both countries. Despite these limitations, our findings provide valuable information on the similar and contrasting views of COVID-19 measures in Japan and the UK.

## Conclusions

Our study revealed similarities and differences in preferences for preventive measures among the respondents from both countries. While both groups agreed on certain PHSM categories (vaccination, working and teaching hours, and COVID-19 testing policy), the responses differed on face-covering rules in public spaces and international and domestic movement restrictions. This indicates that policy to control infection cannot be homogenous across the world. Our findings implicated how policy makers engage in health communication with the public; and for future pandemic preparedness could encourage policy makers to consider regulations which are in alignment with public capacities. Although the specific reasons of similarities and differences were not explored in this study, they warrant future studies to cover various aspects – such as norms, cultures, and the economic and disease burdens of each country – in understanding the public opinions on the PHSM responses.

## Data Availability

The datasets used and analyzed during the current study are available from the corresponding author on reasonable request.

## Abbreviations

FGD: Focus Group Discussion
PCR: Polymerase Chain Reaction
PHSM: Public Health Social Measures
SARS-CoV-2: Severe acute respiratory syndrome coronavirus 2
WHO: World Health Organization
